# Modeling of mold inactivation via cold atmospheric plasma (CAP)

**DOI:** 10.1128/aem.02102-24

**Published:** 2025-04-04

**Authors:** Pavel Demo, Filip Přeučil, Petra Tichá, Mária Domonkos, Eliška Lokajová, Jana Jirešová

**Affiliations:** 1 Department of Physics, Faculty of Civil Engineering, Czech Technical University in Prague 697889 , Prague, Czech Republic; 2 Department of Physics and Measurements, University of Chemistry and Technology Prague 52735 , Prague, Czech Republic; Royal Botanic Gardens, Surrey, United Kingdom

**Keywords:** *Aspergillus brasiliensis*, mycelium inactivation, cold atmospheric plasma, mathematical modeling, growth curves, comparison with experiments

## Abstract

During their reproduction cycles, the omnipresent pathogens produce a broad class of mycotoxins responsible for serious health problems in living organisms. To reduce (or even to eradicate) the microorganisms from the invaded system, various conventional methods are applied in practice, sometimes with counterproductive effects. To overcome these challenges, the cold atmospheric plasma (CAP) is applied to terminate mold proliferation within the system. The paper presents a mathematical model for the elimination of microscopic filamentous types of fungi, specifically molds, by using the CAP. The evolution of mold population is described by a nonlinear logistic equation with a density-dependent inactivation rate. Exactly calculated growth curves are compared with experimental data for *Aspergillus brasiliensis* obtained for two plasma operating times. The results show that if the plasma inactivation rate is comparable to the maximum natural growth rate of the mycelium, the mold colony becomes extinct after a finite time. Otherwise, the mycelium may survive the plasma intervention. The model presented in the paper can be applied to other classes of microorganisms (e.g., bacteria and viruses), using different inactivation techniques (e.g., heating or high pressures with properly defined inactivation rates).

**IMPORTANCE:**

The novelty of this study is to model the extinction process of molds from an invaded system by using a nonlinear logistic equation with a density-dependent inactivation rate. The resulting analytical solution allows us to determine the coverage of the surface by mycelium at arbitrary times. The calculated growth curves are compared with data sets for *Aspergillus brasiliensis*. An advantage of this model is the possibility to obtain relevant information in a matter of minutes, compared to the highly time-consuming real experiments that can take weeks.

## INTRODUCTION

The formation and presence of molds on the surfaces of various materials (food [1, 2], historical artifacts, building materials [3–5], etc.) represent a serious problem in terms of both endangering public health and degrading of materials, resulting in considerable economic and cultural losses. Molds produce mycotoxins (e.g., aflatoxin, coagulase, kinases, collagenase, hemolysins, etc. [6–9]) potentially responsible for serious human and animal health problems.

### Inactivation of molds

To reduce (or even eliminate) pathogens from the infected system, various conventional methods are applied in practice. In particular, in the case of chemical treatment (10–12) of the invaded system, chemical agents with a biocidal effect may have toxic and carcinogenic effects; many of them are now restricted in more than 30 countries (7). In the food industry, the dominant method for mold inactivation is heating a damaged system (13, 14) to sufficiently high (lethal) temperatures, resulting in the deactivation of a large number of undesirable pathogens in food. One of the basic problems of this method is the existence of the so-called heat-resistant molds (15). Their ability to survive these temperatures results in the spoilage of heat-processed food. Additionally, at higher temperatures, nutritional factors of sterilized food usually decrease. The above problems led to an increased interest in replacing conventional methods with innovative treatments of invaded systems by molds lacking the above disadvantages. Cold (non-thermal) atmospheric plasma (CAP) seems to be a promising candidate representing an environmentally acceptable method without leaving any toxic chemical residues, operating at temperatures close to ambient and requiring short processing times (16–20). In general, the synergistic effect of the CAP reactive species (e.g., ozone, singlet oxygen, hydrogen peroxide, hydroxyl radicals, reactive nitrogen compounds, UV radiation, and strong electric fields [21]) is responsible for the lethal effect on the mold and its spores (22).

### Inactivation models

Laboratory tests of mold inactivation are typically long-term experiments (consuming more than 300 h). Therefore, the modeling of this process is beneficial not only for saving time but also for determining straightforwardly the influence of parameters adjustable from the outside on the elimination process.

Regardless of the source of lethal agents (chemical biocides, fungicides, physical plasma, UV radiation [10], and heat [23]), in predictive microbiology, it is usually assumed that the deactivation process follows first-order kinetics (24–26). This approach results in an exponential decrease in the number of microorganisms in the affected sample over time. Alternatively, it can result in linearly decreasing survival curves over time (15). Since one of the important shortcomings of such a linear model is its inability to correctly describe convex or concave survival curves, further models have been developed to cover this gap. In particular, the Weibull distribution and its variations (see, e.g., references 15, 24, and 27–29) and the Gompertz (sigmoid) model in its alternative forms (e.g., references 30–32) belong to those frequently used in microbiology.

The novelty of the model presented in this article is the application of a nonlinear logistic equation with an additional term (representing the density-dependent plasma inactivation rate within a finite time interval) to describe the extinction of mold mycelium in the sample.

## MATERIALS AND METHODS

**Fig 1:**
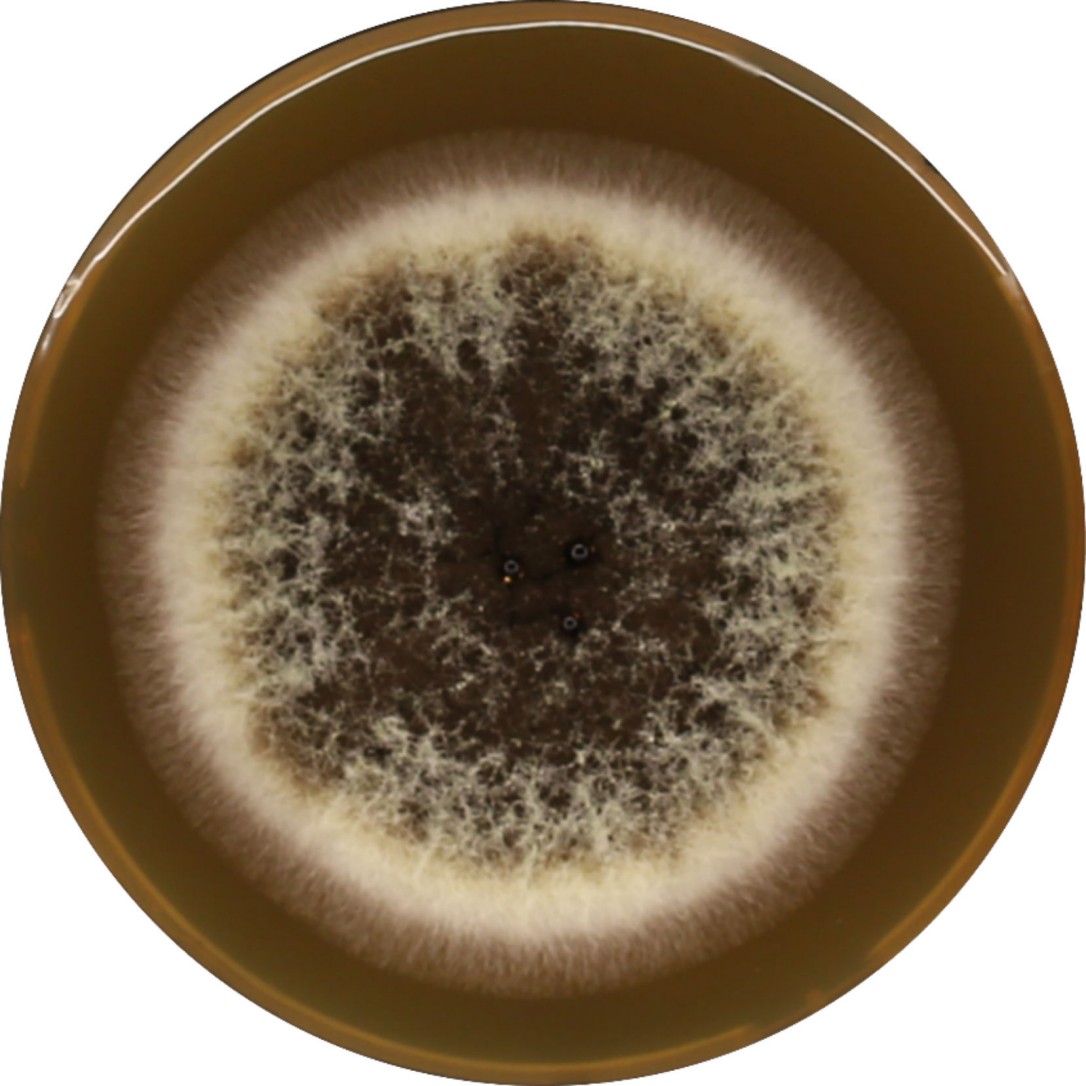
Mold detection on melt extract agar (no plasma treatment).

**Fig 2:**
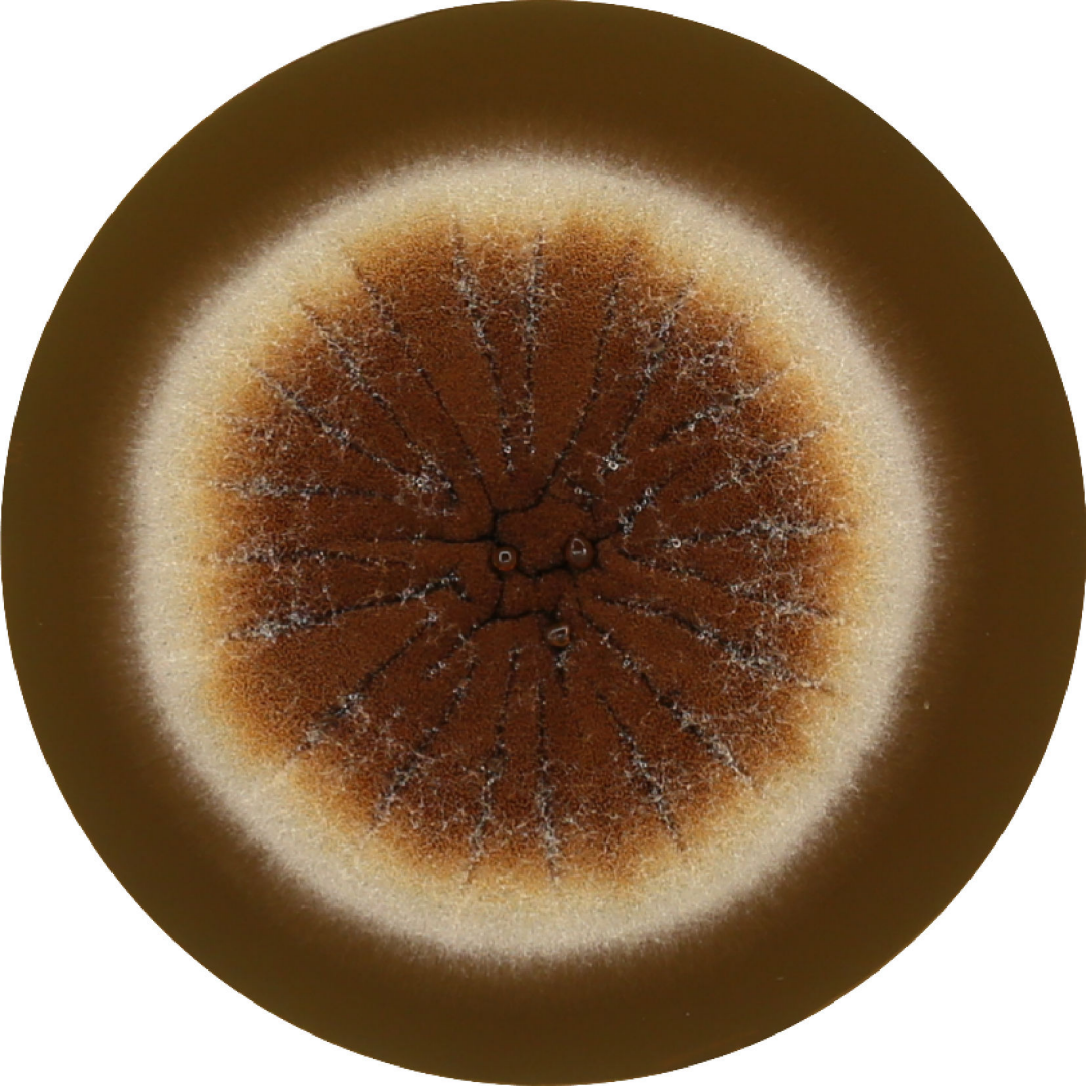
*A. brasiliensis* on melt extract agar treated with diffuse coplanar barrier discharge.

In order to compare experimental data with the theoretical results, the mold *Aspergillus brasiliensis CCM8222* (ATCC 16404) (Fig. 1) was chosen as a suitable candidate for the inactivation procedure (in particular, due to high resolution in coloration between surviving and destroyed parts of mycelium after plasma treatment [Fig. 2]).

*A. brasiliensis* suspension was inoculated in a known concentration onto the surface of melt extract agar (MEA) using three 0.1 mL droplets. Inoculated plates (Petri dishes with a diameter of 90 mm) were placed in an inoculator set to room temperature of 23°C. The inoculated plates were then exposed to CAP produced by applying voltage to accelerate electrons, resulting in partial ionization of the surrounding air between the electrode and the surface of the mycelium.

This process leads to the generation of reactive species (charged and neutral particles, photons) destructively propagating within the mold. The experiments were performed by a diffuse coplanar surface barrier discharge device by Roplass (CZ), with a processing power of 300 W. In the experiments, the samples were treated with CAP for a period of 10 min after a chosen time after inoculation.

The percentage of mold coverage θ was determined using $\theta =\theta_{\text{m}}/\theta_{\text{A}}\times 100\%$ (where $\theta_{\text{m}}$ is the area covered by mold and $\theta_{\text{A}}$ represents the total area). The evaluation of surface mold growth coverage was performed using image analysis. Samples were withdrawn from the climate chamber on a daily basis to assess mold growth. The surface area was detected by capturing high-resolution photographs of the samples using a Canon G1X camera. Subsequently, the images were analyzed using Fiji software, which offers several advantages (simplicity, objectivity, and reproducibility), making it a valuable and widely used tool in research.

Curve fitting was done numerically using SciPy python package function scipy.optimize.curve_fit, which implements the Levenberg-Marquardt algorithm to solve the nonlinear least squares problem. The plots were created using the Matplotlib package.

### Theory and calculation

In this contribution, the mold proliferation over the substrate is characterized by the change in its surface coverage θ with time (see, e.g., references [33–35]). Assume that the surface of the MEA in the Petri dish is inoculated with the spores of an initial coverage $\theta (t=0)=\theta_{0}$.

Since the inactivation mechanism is poorly understood (e.g., reference [24]), it will be assumed, therefore, that the mold elimination occurs via a two-step process:

CAP is activated at $t=t_{0}$, when the part of the substrate is already covered by growing mycelium. From this moment, a variety of lethal agents begin to form inside the thin layer between the electrode and the surface of the mycelium, until the plasma is switched off at $t=t_{1}$. (In our case, $t_{1}-t_{0}=10$ min.)

Newly generated reactive products continuously diffuse within the system to start their killing mission. Damaged mycelium defends against the plasma attack through adaptation mechanisms allowing it to absorb newly established unfavorable environmental conditions. Assume that this revitalization process terminates at $t=t_{2}$, and the surviving part of the mycelium (if any) is prepared from this moment for further proliferation over the MEA substrate. This adaptation mechanism can span between several hours and several weeks (15).

Denoting the plasma inactivation rate by I and assuming the proportional damage of the surface coverage by mycelium, then the balance equation reads


(1) $$\frac{d\theta (t)}{dt}=r\theta (t)\left[1-\frac{\theta (t)}{K}\right]-I\theta (t)[H(t-t_{0})-H(t-t_{2})]$$

with initial condition


(2) $$\theta (t=0)=\theta_{0}.$$

In the above standard logistic equation (36), H(⋅) is the Heaviside generalized function, r represents the proportional increase of the surface coverage (due to the intrinsic metabolism of mold), K stands for the carrying capacity of the system corresponding to the maximum coverage that may be sustained by available resources inside the Petri dish (nutrition, water, living space, etc.), and in the simplest case, we assume I=const. within the time interval $\left\langle t_{0},t_{2}\right\rangle$.

. $0\leq t<t_{0}$. In this case, the exact solution of the equation


(3) $$\frac{d\theta (t)}{dt}=r\theta (t)\left[1-\frac{\theta (t)}{K}\right]$$

with the known initial condition $\theta (t=0)=\theta_{0}$ can be expressed as


(4) $$\theta (t)=\frac{K\theta_{0}}{\theta_{0}+(K-\theta_{0})e^{-rt}}.$$

2. $t_{0}\leq t<t_{2}$. The logistic equation within this time interval has the form


(5) $$\frac{d\theta (t)}{dt}=r\theta (t)\left[1-\frac{\theta (t)}{K}\right]-I\theta (t)$$

with the initial condition


(6) $$\theta (t=t_{0})=\alpha =\frac{K\theta_{0}}{\theta_{0}+(K-\theta_{0})e^{-rt_{0}}}.$$

The solution reads


(7) $$\theta (t)=\frac{\alpha \kappa }{\alpha +(\kappa -\alpha )e^{-(r-I)(t-t_{0})}},$$

where


(8) $$\kappa =\frac{K(r-I)}{r}.$$

3. $t>t_{2}$. The logistic equation has the form (CAP is in the switched-off regime)


(9) $$\frac{d\theta (t)}{dt}=r_{2}\theta (t)\left[1-\frac{\theta (t)}{K}\right],$$

where $r_{2}$ is the intrinsic (natural) growth rate of mycelium. (It is assumed, in general, that $r_{2}$ may differ from r due to possible modification of the substrate by plasma impact).

Analytical solution of equation (9) has the form


(10) $$\theta (t)=\frac{K\beta }{\beta +(K-\beta )e^{-r_{2}(t-t_{2})}},$$

satisfying the initial condition


(11) $$\theta (t=t_{2})=\beta =\frac{\alpha k}{\alpha +(k+\alpha )e^{-(r-I)(t_{2}-t_{0})}},$$

with *α* given by equation (6).

## RESULTS AND DISCUSSION

To test the solutions of appropriate logistic equations with a view to validating the model formulation, two data sets resulting from experiments with *Aspergillus brasiliensis* (inactivated by plasma) were used. The experimental error is less than 4% in all the cases.

**Fig 3:**
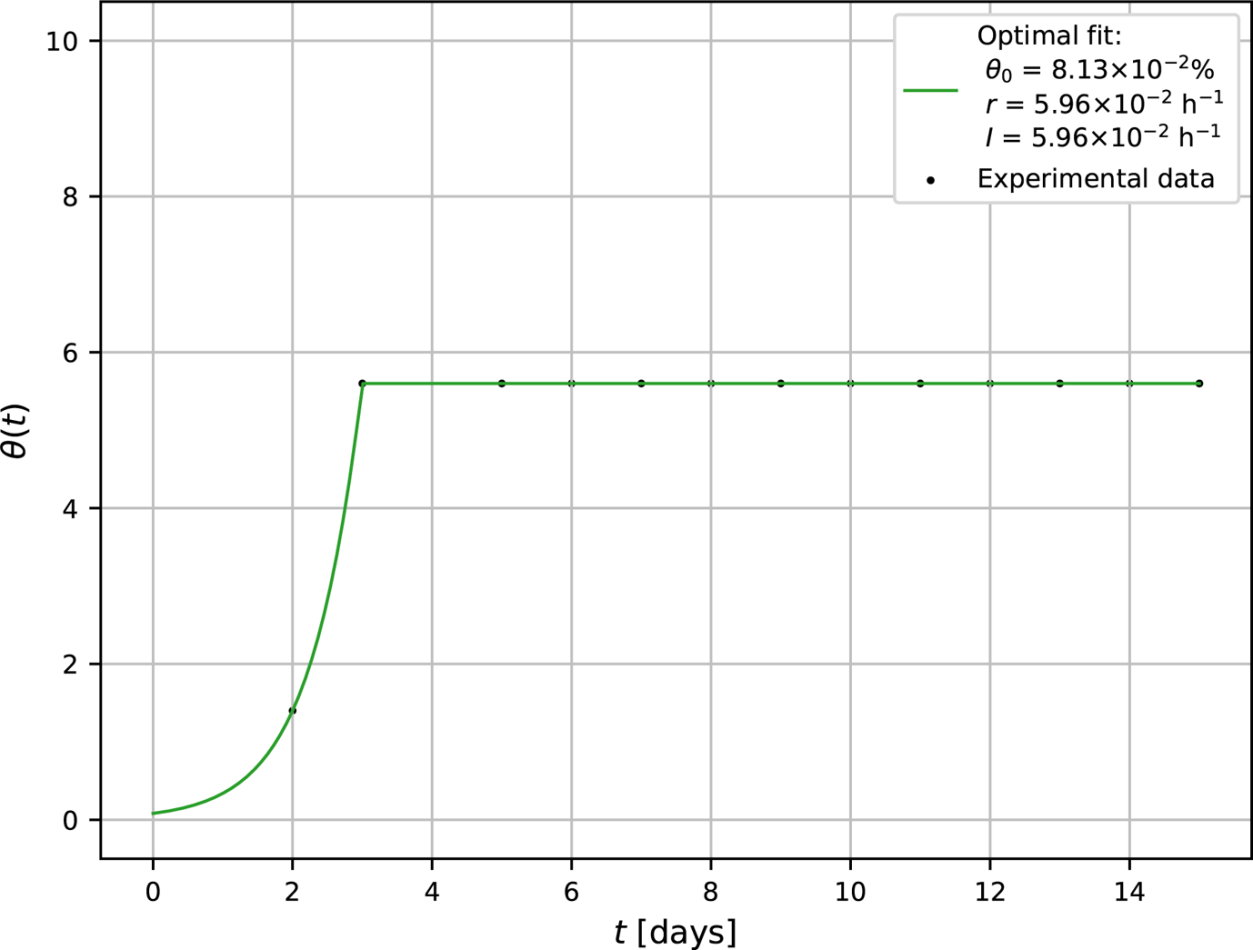
Growth curve of *A. brasiliensis* after plasma intervention at *t*_0_=72 h after inoculation.

The probability of mold survival after plasma intervention was studied in two operating modes. In the first experiment, the plasma was activated at $t_{0}=72$ h after initial inoculation of the substrate and with operating time $\Delta t=t_{1}-t_{0}=10$ min. In order to determine the intrinsic (natural) growth rate r, consider the experimentally obtained points X=[48h,1.4%] and Y=[72h,5.6%] lying on the exponential part of the growth curve (Fig. 3). Inserting X and Y into solution (4), $\theta_{0}$ and r can be readily calculated to be $\theta_{0}=0.0813\%$ and $r=0.0596{\text{h}}^{-1}$.

On the other hand, since the growth curve remains unchanged for $t>t_{0}=72$ h, it means that the plasma attack terminates further proliferation of the mycelium through the substrate. Since the extinct mold is not removed from the system, the amount of destroyed mycelium remains constant, $\theta (t>t_{0})=\alpha$. Following solution equation (7), this situation occurs if I=r. Consequently, the devastating effect of CAP is comparable to the energy of the metabolic process governing the natural growth rate.

**Fig 4:**
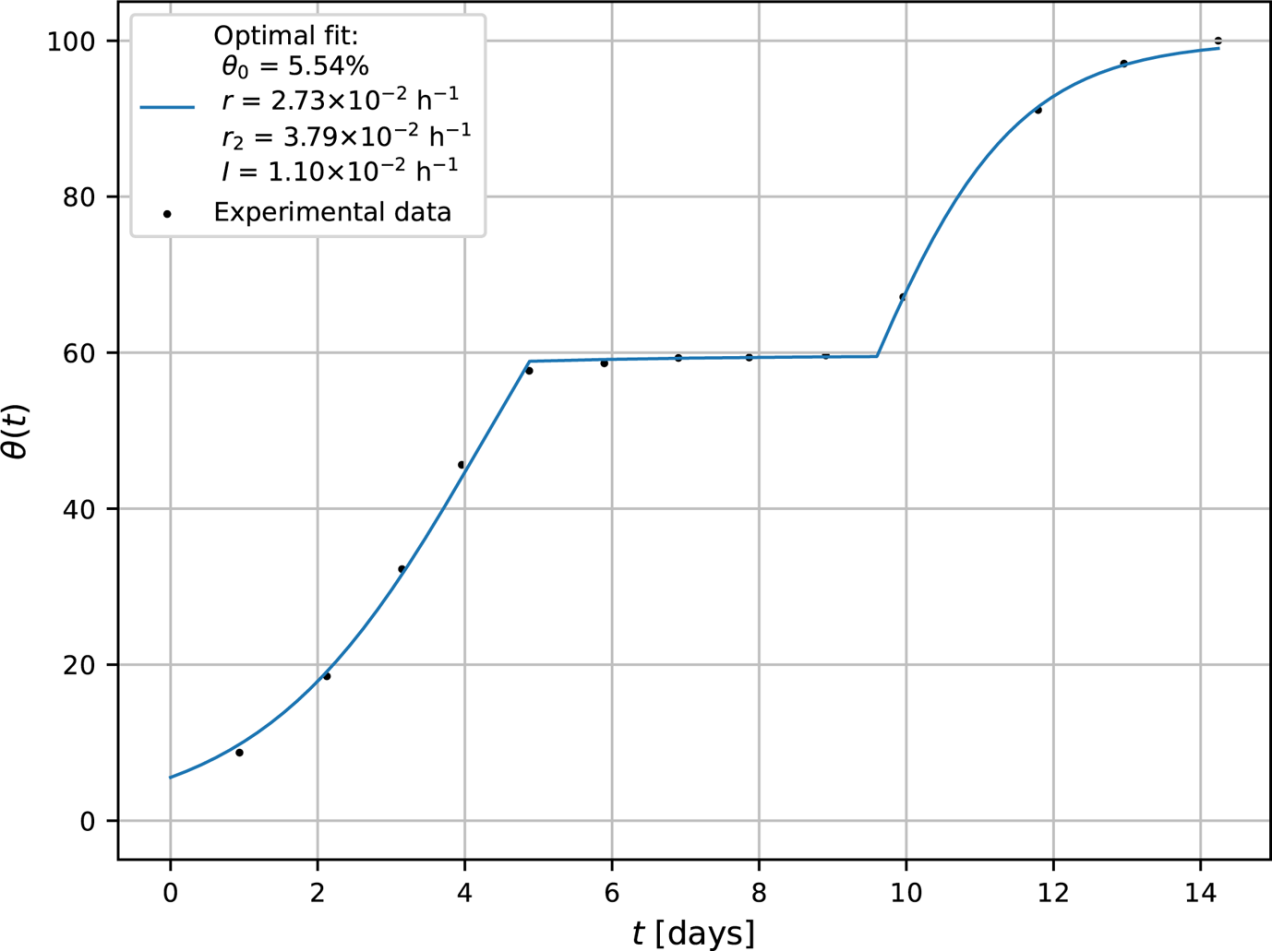
Modeling of the growth curve of *A. brasiliensis* with plasma activated at 117 h after inoculation.

The presented model was also applied to the reproduction of *A. brasiliensis* growth when plasma begins to act after $t_{0}=117$ h after MEA inoculation (see Fig. 4).

Also, in this case, the growth curve remains approximately constant within time interval ⟨117h,230h⟩ followed by further growth with different natural growth rate $r_{2}$ (see Fig. 4). This stagnation period reflects the revitalization regime of damaged mycelium (regime analogous to the lag period in the growth of molds under normal condition). During Δt=113h, the intrinsic metabolic processes are dominantly oriented to the adaptation of surviving mycelium to abruptly changed boundary conditions imposed by plasma intervention.

### Conclusions

The principal conclusions drawn from the results presented in this study are as follows:

1. Analytical solution of the logistic equation with a density-dependent inactivation rate allows to determine the coverage of the substrate by mycelium at arbitrary time together with the natural growth rate.
2. In this study, the constant inactivation rate was considered in order to simplify appropriate analytical calculations. Consequently, the future extension to overcome this limitation includes the precise modeling of the inactivation term.
3. One of the advantages of the presented model is that while real experiments are highly time-consuming (taking weeks), this approach allows, at least, to obtain related information in a matter of minutes.
4. The model will also be applied in real-world scenarios, particularly modeling the inactivation of molds on surfaces of building materials (such as wallpaper, plaster, plasterboard, hardboard, etc.) in the near future.

## Data Availability

Data will be made available on request.
